# Association Between Procalcitonin and Post-hepatectomy Liver Failure in Hepatocellular Carcinoma Patients

**DOI:** 10.3389/fphar.2021.791322

**Published:** 2021-11-18

**Authors:** Yi-Ran Li, Xiao-Yan Meng, Rui-Qing Zong, Fei-Xiang Wu

**Affiliations:** Department of Intensive Care Medicine, Eastern Hepatobiliary Surgery Hospital, The Third Affiliated Hospital of Naval Medical University, Shanghai, China

**Keywords:** procalcitonin, post-hepatectomy liver failure, hepatocellular carcinoma, biomarker, mortality

## Abstract

**Objectives:** Procalcitonin (PCT) has long been proved as an early diagnostic signal for postoperative outcomes. The purpose of this study is to explore the value of serum procalcitonin levels in predicting post-hepatectomy liver failure (PHLF), and further to declarethe relationship between postoperative PCT and short-term prognosis in patients after hepatectomy.

**Methods:** Clinical data of patients with hepatocellular carcinoma (HCC) who underwent hepatectomy from June 1st, 2019 to September 31st, 2020 at Shanghai Eastern Hepatobiliary Surgery Hospital had been retrospectively analyzed. Logistic regression analysis was used to evaluate the risk factors related to PHLF. The Kaplan-Meier method was used to calculate the PHLF rate and 30-day survival after surgery.

**Results:** A total of 885 patients with complete data were finally included in analysis, 311 of them with elevated serum PCT (≥1 ng/ml). Results of the logistic regression analysis suggested a significant association between PCT and PHLF [HR, 95%CI; 3.801 (1.825, 7.917), *p* < 0.001]. Other significant risk factors for PHLF included portal hypertension, portal blocking time (>30 min) and blood transfusion (>200 ml). Kaplan-Meier analysis also suggested a higher PHLF rate in elevated PCT patients [9.0% (95% CI, 7.3 to 12.8 VS. 1.9% (95% CI, 1.1–4.3)); *p* < 0.001]. For secondary outcomes, elevated PCT was also highly associated with postoperative sepsis, ICU admission, 30-day mortality and 3-month mortality.

**Conclusion:** Elevated procalcitonin level in patients after hepatectomy is related to higher PHLF rate, with lower 30-day survival and poor short-term postoperative outcomes.

## Introduction

Procalcitonin (PCT) is a prohormone of 116-amino acid peptides (Maruna et al., 2000). In healthy individuals, PCT is produced by thyroidal and adipose tissue, and could further be cleaved to form calcitonin for maintaining serum calcium homeostasis. The normal serum value of PCT is < 0.05 ng/ml, while a higher serum level of PCT could be a sign of a serious bacterial infection (Meisner, 2002). Thus, PCT has been deemed as an infection biomarker that can effectively guide the use of antibiotics in critically ill patients, while more literature has reported that elevated PCT levels could be associated with other long and short-term outcomes after surgery (Sponholz et al., 2006; Engel et al., 2013). For instance, PCT levels can be elevated in patients with hepatitis, cirrhosis and liver failure.

Liver resection is an effective treatment for primary liver cancer. Postoperative monitoring of PCT can guide the use of antibiotics, and be further applied to control postoperative infections and to reduce postoperative complications as well (Jones et al., 2007). PCT could rise within 4 h after infection or injury, and drop back to baseline in 2–3 days, this character makes it a sensitive indicator for clinical use in disease detection. Previous literature reports that PCT is acceptable for predicting infection in patients with liver disease (Lin et al., 2014). At present, there are few reports have detected the value of PCT in indicating outcomes after hepatectomy, and the diagnostic cutoff value of PCT in liver diseaseremains controversial (Jody et al., 2015).

The present study aims to determine whether postoperative serum PCT level is associated with and post-hepatectomy liver failure (PHLF) and other short-term outcomes in patients diagnosed with hepatocellular carcinoma (HCC) after hepatectomy.

## Materials and Methods

### Patients and Methods

We retrospectively identified all patients who underwent hepatectomy from June 1st, 2019 to September 31st, 2020 at Shanghai Eastern Hepatobiliary Surgery Hospital. Patients were included using the following inclusion criteria: (a) patients with HCC who received hepatectomy; all diagnoses were confirmed by pathological examination of the surgical specimen; (b) age of 18–80 years; and (c) Child-Pugh A or B. Exclusion criteria were as follows: (1) patients with a history of any other malignant tumor before hepatectomy; (2) without or unavailable postoperative PCT data; (3) incomplete data recording of postoperative outcome; (4) without informed consent. This study was approved by the local ethics committee. Written informed consent was obtained from all participants.

### Data Collection

All data were based on hospital medical records and clinical follow-up, the last follow-up data were January 31, 2021. The primary outcome of this study was the rate of PHLF. Liver failure was defined as a total bilirubin level of ≥5 mg/dl or prothrombin time of <40% due to exacerbation of the background liver disease.

Secondary outcomes include postoperative sepsis/septic shock, ICU admission, other organ dysfunctions except for liver, reoperation for any surgical adverse event, 30-day mortality and 3-month mortality.

The serum PCT levels were measured in serum samples. Samples were acquired at 12 h after surgery and then stored at −80°C using an automated PCT immunoassay (Elecsys BRAHMS PCT; Roche Diagnostics GmbH, Mannheim, Germany). The detection limit was 0.01 ng/ml.

### Statistical Analysis

Continuous variables were presented as mean ± SD or median with IQR (interquartile range). Categorical variables were grouped based on clinical findings, presented as counts and percentages. Patients were divided into two groups according to the measured PCT. A measured serum PCT value >1 ng/ml was defined as elevated PCT according to previous researches, while each measured PCT value was also applied in the analysis as additional evidence. Student t test, Fisher exact test or Chi-squared test were used accordingly. A univariate logistic regression model for all variables potentially associated PHLF has been applied, then a multivariable analysis was further applied using selected variables with *p* < 0.1in the univariate analysis, using a backward stepwise selection process. The Kaplan-Meier method was used to calculate the recipient and graft survival, and subgroup analysis was then applied according to the risk factors. The significance level was set as *p* < 0.05 at two tails. All statistical analyses were performed using the SPSS version 25.0 software program (IBM SPSS, Chicago, IL) and R version 4.0.3 software (Institute for Statistics and Mathematics, Vienna, Austria; http://www.r-project.org/).

## Results

**FIGURE 1:**
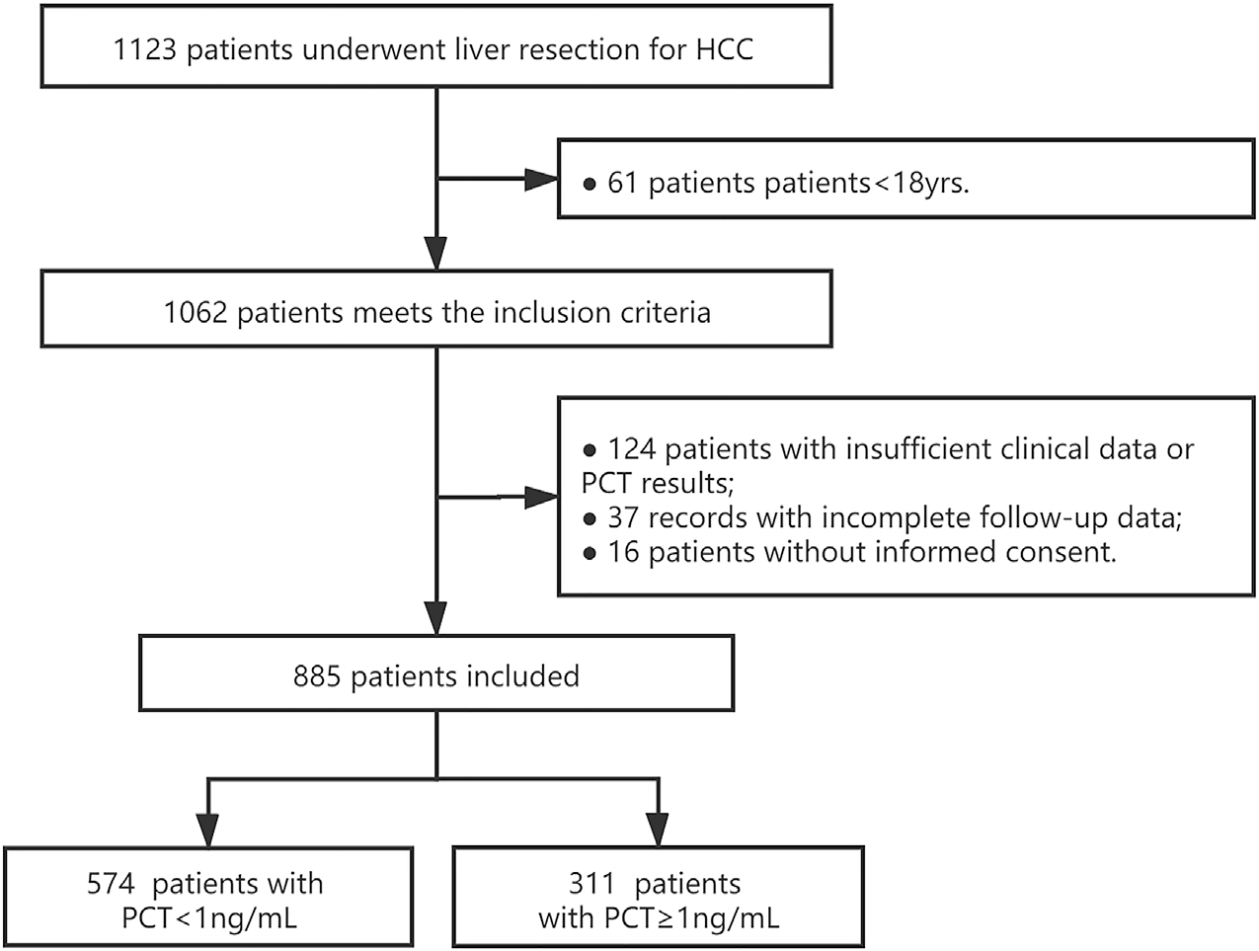
Study flow. PCT: procalcitonin; HCC: hepatoma carcinoma cell.

From June 1st^,^2019 to September 31st^,^ 2020, a total of 1,123 patients underwent liver resection for HCC in Eastern Hepatobiliary Surgical Hospital. After screening, 885 of them were feasible for analysis with sufficient clinical data and assigned informed consent. After PCT measurement, 574 patients had a measured serum PCT level of less than 1 ug/mL, while 311 of them with comparatively higher serum PCT (≥1 ug/mL). The study flow was presented in Figure 1. The baseline characteristics and operative variables of the patients in the training cohort were listed in Table 1. Similar results were presented in most baseline variables, however, in terms of disease-related or operative variables, significant differences existed in DM, tumor number, tumor size, portal hypertension, ascites, blood loss (over 500 ml or less), blood transfusion (over 200 ml or less) and liver resection (over three liver segments or less).

**TABLE 1:** Baseline Characteristic and operative variables.

Variable No. (%)	PCT<1 ng/ml (N = 574)	PCT≥1 ng/ml (N = 311)	*p* Value
PCT, median (IQR), ng/mL	0.13 (0.29, 0.59)	1.1 (1.0, 5.9)	-
Gender, female	117 (20.4)	63 (20.3)	0.965
Age,mean (SD),yr	55.5 ± 11.6	57.5 ± 12.2	0.227
Hypertension	129 (22.5)	83 (26.7)	0.226
DM	59 (10.3)	46 (14.8)	0.048
Smoking history	238 (41.5)	125 (40.2)	0.714
Drinking history	184 (32.1)	91 (29.3)	0.391
Tumor number, multiple	89 (15.5)	62 (19.9)	0.094
Tumor size, >5 cm	231 (40.2)	164 (52.7)	<0.001
Antivirus	209 (36.4)	107 (34.4)	0.632
HBV-DNA, ≥50IU/m	249 (43.4)	141 (45.3)	0.575
Cirrhosis	274 (47.7)	158 (50.8)	0.383
Portal hypertension	102 (17.8)	71 (22.8)	0.070
Ascites	54 (9.4)	46 (14.8)	0.016
Portal blocking time,>30min	133 (232.2)	80 (37.6)	0.396
Blood loss,>500 ml	113 (19.7)	102 (32.8)	<0.001
Blood transfusion,>200 ml	103 (17.9)	107 (34.4)	<0.001
Liver resection range, ≥3 segment	172 (30)	117 (37.6)	0.025
Peritoneoscope	16 (2.8)	8 (2.6)	0.851

Abbreviation: PCT: procalcitonin; SD, standard deviation; DM, diabeties mellitus.

**FIGURE 2:**
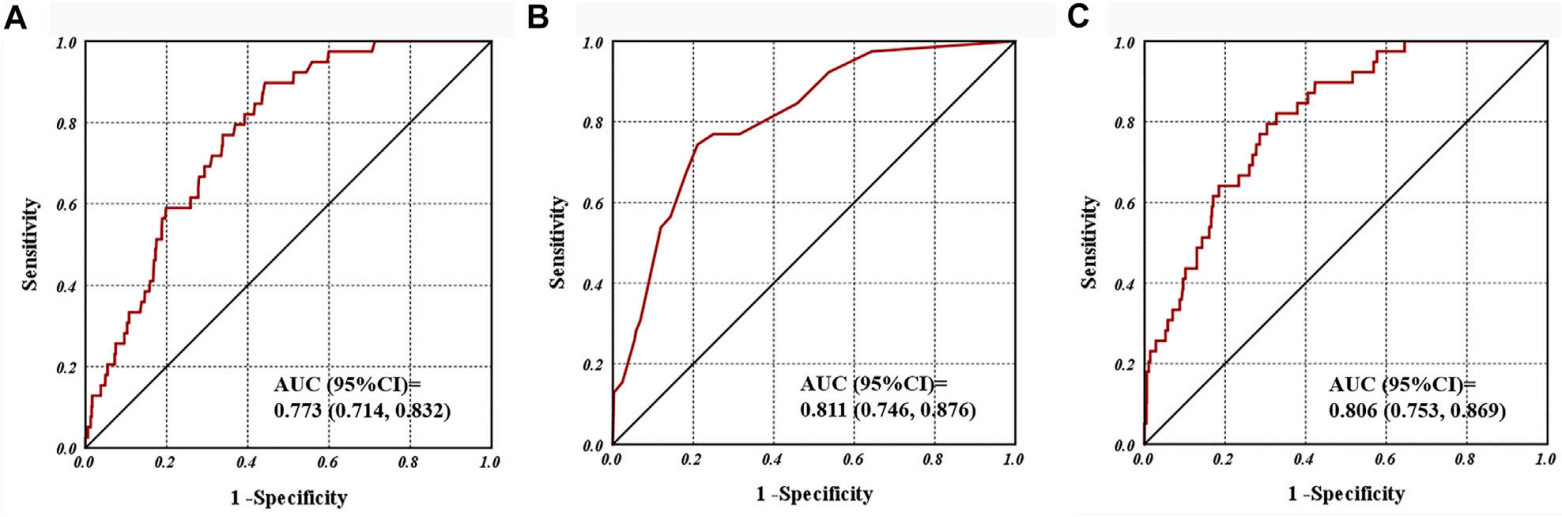
Receiver operating characteristic curves for **(A)** post-operative serum procalcitonin; **(B)** model 1; **(C)** and model 2 associated with post-hepatectomy liver failure.

For the primary outcome, a total of 39 patients had been diagnosed of PHLF, among them, 28 patients have a measured PCT≥1 ng/ml. In the univariate analysis, nine variables were screened out as potential risk factors for PHLF with unadjusted *p* value < 0.1, there were elevated serum PCT level (measured serum PCT value as well), cirrhosis, ascites, portal hypertension, tumor number, blood loss, blood transfusion and liver resection range (Table 2). Further, we applied these nine variables into a multivariable analysis, ultimately four variables were included in the model (Table 3). Both of categorical variable of PCT (≥1 ng/ml) and measured value of PCT exhibited significant relation with the PHLF events [HR, 95%CI; 3.801 (1.825, 7.917), *p* < 0.001; 1.05 (1.017, 1.085), *p* = 0.003]. Other Signiant risk factors included portal hypertension, portal blocking time (>30 min) and blood transfusion (>200 ml) (Table 3). Besides, the ROC curves for serum value of PCT, model one and model two of multivariate logistic regression analysis was presented in Figure 2, a promising predictive value of them for PHLF was revealed.

**TABLE 2:** Univariate and multivariate analyses for risk factors associated with PHLF.

Variable	Hazard ratio (95% CI)	*p* Value
PCT (≥1 vs. <1 ng/ml)	5.064 (2.485, 10.319)	**<0.001**
PCT*	1.092 (1.057, 1.129)	**<0.001**
Sex (male vs. female)	0.435 (0.153, 1.240)	0.119
Age (year)	0.989 (0.964, 1.015)	0.414
Hypertension	1.059 (0.659, 1.703)	0.812
DM	1.353 (0.552, 3.316)	0.509
Smoking history	1.025 (0.465, 2.262)	0.951
Drinking history	1.729 (0.777, 3.843)	0.179
Surgical history	0.68 (0.159, 2.901)	0.602
Cirrhosis	2.45 (1.225, 4.900)	**0.011**
Portal hypertension	3.053 (1.576, 5.913)	**0.001**
Ascites	2.115 (0.944, 4.739)	**0.069**
Tumor number (multiple vs single)	3.664 (1.886, 7.117)	**<0.001**
Tumor size (>5 vs. ≤5, cm)	1.066 (0.56, 2.030)	0.845
Portal blocking time (>30 vs. ≤30, min)	2.563 (1.334, 4.922)	**0.005**
Blood loss (>500 vs. ≤500, ml)	3.921 (2.048, 7.508)	**<0.001**
Blood transfusion (>200 vs. ≤200, ml)	5.677 (2.919, 11.041)	**<0.001**
Liver resection (≥3 vs. <3 segment)	1.784 (0.941, 3.380)	**0.076**
Antivirus	0.607 (0.292, 1.262)	0.182
HBV DNA (≥50 vs. <50 IU/ml)	1.216 (0.64, 2.312)	0.55

The bold values represent the statistical significance of the data.

**TABLE 3:** Multivariate analyses for risk factors associated with PHLF.

Variable	Hazard ratio (95% CI)	*p* Value
Model 1		
PCT	3.801 (1.825, 7.917)	0.000
Portal hypertension	2.973 (1.473, 6.003)	0.002
Portal blocking time (>30 vs. ≤30, min)	2.369 (1.181, 4.751)	0.015
Blood transfusion (>200 vs. ≤200, ml)	3.937 (1.973, 7.853)	0.000
Model 2		
PCT*	1.05 (1.017, 1.085)	0.003
Portal hypertension	3.305 (1.634, 6.683)	0.001
Portal blocking time (>30 vs. ≤30, min)	2.639 (1.307, 5.327)	0.007
Blood transfusion (>200 vs. ≤200, ml)	4.246 (2.13, 8.464)	0.000

**FIGURE 3:**
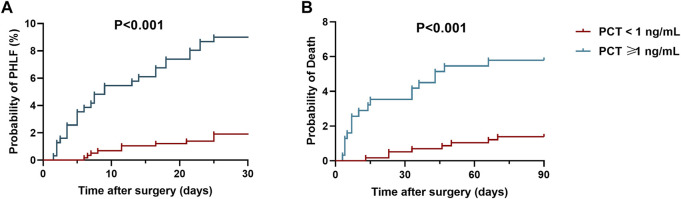
Kaplan-Meier analysis for post-operative procalcitonin on **(A)** post-hepatectomy liver failure rate; **(B)** 3-month mortality.

In terms of secondary outcomes, an unadjusted logistic regression analysis suggested that a postoperative serum PCT value over 1 ng/ml was significant associated with postoperative sepsis [HR, 95%CI; 7.398 (4.921, 11.121); *p* < 0.001], ICU admission [HR, 95%CI; 4.346 (1.868, 10.115); *p* = 0.001], 30-day mortality [HR, 95%CI; 6.979 (1.932, 25.207); *p* = 0.003] and 3-month mortality [HR, 95%CI; 4.976 (2.055, 12.049); *p* < 0.001]. No significance has been found between PCT with severe adverse events or reoperation (Table 4). Kaplan-Meier analysis was also applied for analysis of elevated serum PCT level (>1 ng/ml) with PHLF and mortality. The results showed that, compare with PCT less than 1 ng/ml, an elevated serum PCT level (>1 ng/ml) was associated with a higher PHLF rate ([9.0% (95% CI, 7.3 to 12.8 VS. 1.9% (95% CI, 1.1–4.3)); *p* < 0.001, Figure 3A]). Besides, elevated serum PCT level (>1 ng/ml) was also associated with a higher 3-month mortality rate ([5.8% (95% CI, 2.6 to 9.5 VS. 1.2% (95% CI, 1.0–2.3); *p* = 0.001, Figure 3B]).

**TABLE 4:** Secondaryoutcomes.

	PCT<1 ng/ml (N = 574)	PCT<1 ng/ml (N = 311)	Hazard ratio (95% CI)	*p* Value
Sepsis/septic shock (%)	37 (6.4)	105 (33.8)	7.398 (4.921, 11.121)	<0.001
ICU admission (%)	8 (1.4)	16 (5.5)	4.346 (1.868, 10.115)	0.001
Organ disfunction^a^ (%)	33 (5.7)	21 (6.8)	1.187 (0.674, 2.093)	0.552
Reoperation (%)	6 (1.0)	8 (2.7)	1.862 (0.596, 5.824)	0.285
30-day mortality (%)	3 (0.5)	11 (3.5)	6.979 (1.932, 25.207)	0.003
3-month mortality (%)	7 (1.2)	18 (5.8)	4.976 (2.055, 12.049)	<0.001

a Other organ disfunction except for liver. Abbreviation: PCT, procalcitonin; CI: confidence interval.

## Discussion

PHLF is the most important cause of morbidity after hepatectomy. Early diagnosis and prompt treatment of postoperative infection can improve the prognosis of patients. Therefore, we need a clinical diagnostic index that can predict PHLF after hepatectomy and prepare ahead of schedule for more intensive medical care (Angele and Faist, 2002; Owens and Stoessel, 2008). According to previous researches, PCT levels are elevated in bacterial, parasites, and infections, and the PCT is suitable for the diagnosis of infection. The early use of antibiotics is emphasized in the Sepsis Guidelines, which is closely related to the mortality rate of patients. As a useful biomarker, PCT can even distinguish some severe complications after surgery.

For surgical patients with pre-existing liver diseases or scheduled for hepatectomy, PCT could also be valuable in anticipating surgical outcomes. Patients with liver disease, especially cirrhosis, are prone to bacterial infection, thus early diagnosis of infection in patients with underlying liver diseases is necessary (Elefsiniotis et al., 2006). Besides, in 2 days after abdominal surgery, the value of PCT is increased due to bile leakage of residual liver, causing abdominal bacteria pollution, which may lead to local or systemic infections (Meisner et al., 1998; Jekarl et al., 2013). An accurate PCT cut-off value for postoperative outcomes is very important to avoid overtreatment or misdiagnosis. It is well known that the PCT cut-off value of 0.5 ng/ml was considered significant for sepsis diagnosis. Previous research, the PCT concentration ≥1.8 ng/ml for two consecutive days has predictive significance with best sensitivity and specificity for severe postoperative adverse events (Rau et al., 2000; Mofidi et al., 2009; Adachi et al., 2015; Chen et al., 2017). The half-life of serum procalcitonin was 20–24 h, this study chose a time point of PCT on the first day after surgery as the research time. The previous literature also reports that a PCT value ≥ 0.5 ng/ml may indicate that this insult still exists in the possible evolution of postoperative infections after 24 h of surgery. However, non-infectious factors after the operation cause the PCT value to increase, and it is difficult to find a suitable PCT cut-off value to diagnose other surgical complications. Previous literature has shown that a PCT cut-off value of ≥1 ng/ml can diagnose infection on the first day after surgery (Mokart et al., 2005a; Mokart et al., 2005b).

In the present study, we determined that elevated serum PCT levels (>1 ng/ml) are independently associated with increased incidence of PHLF in patients who underwent hepatectomy for HCC. As the importance of diagnostic clinical algorithm in decision-making in HCC patients, Barcelona Clinic Liver Cancer (BCLC) was to analyze the possible prognostic factors of the outcome (Llovet et al., 1999; Trovato et al., 2013). PCT value could be used for example to implement the BCLC and to predict PHLF and short-term outcomes after hepatectomy for HCC patients. However, the reasons underlying the correlation of PCT value and PHLF remain unclear. Our findings also demonstrated that increased serum PCT levels were present in patients with several of the complications including portal hypertension, ascites and larger tumor size. These results are in line with previous reports that the serum PCT levels were higher in the patients with alcoholic liver cirrhosis and portal hypertension due to various digestive diseases. Besides, Evidence has proved that portal hypertension could worsen bacterial translocation by increasing intestinal permeability, while other findings have shown that patients with cirrhosis and ascites have higher rates of bacterial infections than patients with nonalcoholic liver disease (Rau et al., 2000; Chen et al., 2017). These pieces of evidence suggest that bacteremia, or even sepsis, is common in patients with elevated PCT, and severely infected patients are at high risk for PHLF. Thus, the existence of bacteremia or even sepsis might play a core role in those PHLF patients with elevated PCT. However, the patient’s background varies based on the cause of hospitalization and etiology, therefore, it might be difficult to implicitly conclude as serum PCT levels differ based on the cause of infection. Specially, the reason for the elevated serum PCT levels in patients without bacterial infection remains unclear.

In our study, increased PCT value is also seen in other complications of hepatectomy besides PHLF. First of all, the increased incidence of sepsis or septic shock is higher in patients with elevated PCT. Besides, results also show that patients with elevated PCT values have higher 30-day mortality as well as 3-month mortality, with more risks for ICU admission. These results are in line with conclusions from previous studies that PCT assay may help identify complications in patients after hepatectomy, and predict short-term prognosis. For instance, the PCT value is elevated in complications of liver cirrhosis. Furthermore, PCT assessment may potentially help physicians to limit the number of prescriptions for antibiotics. Other diseases associated with elevated PCT include cardiogenic shock, severe pancreatitis, severe renal dysfunction, ALF, liver transplantation in children, and these diseases all suggest a poor prognosis (Rey et al., 2007; Minami, 2014; Patout et al., 2014; Sugihara et al., 2017; Tschiedel et al., 2018). Previous studies have also indicated that an elevated PCT value is associated with complications and poor prognosis after hepatectomy, yet its underlying mechanism remains unknown (Kretzschmar et al., 2001).

Several limitations of this study should be mentioned. Firstly, this is a retrospective study involving a relatively small sample size; therefore, a multi-center patient cohort is needed for further validation of our results. Second, several potential risk factors for PHLF are not analyzed in this trial, including serum CPR level and tumor markers. Besides, the causes of elevated PCT in patients with poor prognosis have not been investigated in this research.

## Conclusion

In conclusion, we determined that an elevated serum PCT level of patients after hepatectomy has a high predictive value for PHLF, as well as other severe postoperative short-term outcomes, including mortality and ICU admission. Post-operative serum PCT could be an effective biomarker for the prediction of outcomes and instruction of further intensive medication.

## Data Availability

The raw data supporting the conclusion of this article will be made available by the authors, without undue reservation.
